# Association between Lower Urinary Tract Symptoms and Sleep Quality of Patients with Depression

**DOI:** 10.3390/medicina57040394

**Published:** 2021-04-19

**Authors:** Mikolaj Przydacz, Michal Skalski, Jerzy Sobanski, Marcin Chlosta, Karol Raczynski, Katarzyna Klasa, Dominika Dudek, Piotr Chlosta

**Affiliations:** 1 Department of Urology, Jagiellonian University Medical College, 30-688 Krakow, Poland; marcin.p.chlosta@gmail.com (M.C.); raczynski.karol@gmail.com (K.R.); piotr.chlosta@gmail.com (P.C.); 2 Department of Adult Psychiatry, University Hospital, 31-501 Krakow, Poland; lekmskalski@gmail.com; 3 Department of Psychotherapy, Jagiellonian University Medical College, 31-138 Krakow, Poland; molocko@poczta.fm (J.S.); katarzynaklasa@poczta.onet.pl (K.K.); 4 Department of Affective Disorders, Jagiellonian University Medical College, 31-501 Krakow, Poland; dominika.dudek@poczta.fm

**Keywords:** lower urinary tract symptoms, sleep, depression

## Abstract

*Background and Objectives*: In the general population, sleep disorders are associated with lower urinary tract symptoms (LUTS) including urinary incontinence (UI). This connection has not been explored fully in specific patient groups. Thus, we investigated the association between sleep quality and LUTS for patients with depression. *Materials and Methods:* This study was prospective and cross-sectional. We analyzed questionnaire data on depression, sleep quality, LUTS, and UI from depressed patients treated in our department of adult psychiatry. We used the Hamilton Rating Scale for Depression, the Holland Sleep Disorders Questionnaire, the International Prostate Symptom Score, and the International Consultation on Incontinence Questionnaire-Short Form. *Results:* In total, 102 patients treated for depression were enrolled. We found a statistically significant correlation between depression severity and sleep quality. A significant correlation was also investigated for sleep quality and LUTS severity. The group of depressed patients with moderate or severe LUTS had greater sleep problems compared with patients who had mild urinary tract symptoms or no symptoms. With regression analysis, we further demonstrated that the relationships between LUTS and sleep quality as well as UI and sleep quality in depressed patients are independent from age and sex. *Conclusions:* In the cohort of patients treated for depression, sleep quality correlated with LUTS including UI. We suggest that the negative effect of LUTS and UI on sleep quality that we observed should lead to the re-evaluation of current recommendations for diagnosis and treatment of sleep problems among patients with depression.

## 1. Introduction

Lower urinary tract symptoms (LUTS) include storage, voiding, and postmicturition symptoms [1]. LUTS are highly prevalent and costly conditions worldwide [2], and they have a significant negative impact on quality of life [3].

There are many reports of significant relationships between LUTS and sleep quality [4,5,6,7]. These studies have measured LUTS as a risk factor for severe sleep disturbance. The most extensively examined condition was nocturia, the complaint of an individual who must wake at night to void. Among all LUTS, nocturia was suggested to have the strongest negative impact on sleep quality [7]. Repeated fragmentation of sleep due to nocturia further results in daytime drowsiness, poor concentration, and anxiety. These factors lead to problems in occupational functioning and physical and emotional health, thus affecting quality of life [8]. Other LUTS associated with storage and voiding have also been shown to be independent predictors of sleep dysfunction [4,5,6,7]. Despite these several studies, comorbid sleep disturbances and LUTS, particularly nocturia, remain underreported and undertreated, which leads to a considerable burden and major public health issue (i.e., both conditions are highly prevalent) due to their consequences on quality of life [9]. Further, all past studies have focused on only the general population (i.e., mentally healthy persons without diagnosis of depression or other psychiatric disorders). The association between LUTS and sleep quality has not been studied sufficiently for specific patient populations.

Patients with depression represent a unique cohort because of the bidirectional nature of the relationship between depression and LUTS [8,10]. Therefore, it has been recommended that psychiatrists and urologists should jointly devote attention to comorbid LUTS and depression to encourage interdisciplinary treatment approaches. Still, for depressed patients, there are no published data regarding the relationship between LUTS and sleep quality, although sleep problems are common in this patient population. These data are necessary to provide a comprehensive understanding of the medical workup and integrated care that such patients need. For the general population, it has been recommended that clinicians consider the assessment of LUTS in patients with sleep disorders because LUTS may emerge from or intensify sleep problems [4]. In depressed patients, it is not known whether LUTS have an impact on sleep quality. Moreover, sleep quality in depressed patients may be independently decreased by depression itself. The link between LUTS and sleep disorders in specific patient groups has been gaining attention [9]. This new focus on LUTS is also related to significant effects of multiple and diverse conditions, for instance, pain or diarrhea, on sleep quality [11,12,13]. Therefore, we investigated the connections between LUTS and sleep quality in patients with depression. By analyzing the designs of large-scale studies of the general population and specific patient groups (e.g., shift workers) [4,5,14,15], we hypothesized that depressed patients who report LUTS experience greater impairment of sleep compared with depressed patients lacking LUTS.

## 2. Materials and Methods

We prospectively included depressed patients treated between 2014 and 2015 in our outpatient and inpatient department of adult psychiatry at the University Hospital in Krakow, Poland. All included patients met both DSM-5 and ICD-10 criteria for depression, and psychiatrists established their diagnoses.

### 2.1. Instruments

The Hamilton Rating Scale for Depression (HRSD) was used to classify depression severity. The instrument provides an indication of depression and information on depression severity and may serve as a guide to evaluate recovery [16]. The HRSD consists of 17 questions with a total score between 0 and 54. For this study, patient scores were classified as in remission—no depression (0–7), with mild depression (8–16), with moderate depression (17–23), and with severe depression (≥24). Psychiatrists completed the HRSD questionnaire.

The Holland Sleep Disorders Questionnaire (HSDQ) was used to assess sleep quality. The instrument differentiates patients with a clinically diagnosed sleep disorder from individuals without sleep complaints [17]. The HSDQ generates a global sleep disorder score and may further differentiate 6 categories of sleep disorders. The HSDQ contains 32 questions. The total score is between 32 and 160, with a higher score indicating lower sleep quality. From the HSDQ, we focused on the continuous variable of general sleep disorder. The questionnaire was self-administered by patients.

The International Prostate Symptom Score (IPSS) was used to assess the severity of LUTS. IPSS contains 7 questions related to storage, voiding, and postmicturition symptoms. Because of its simplicity and the versatility and reliability in assessing the severity of LUTS, the IPSS is also used for women. The IPSS scale served as a reference instrument in studies of the association of LUTS and depressive symptoms in the general population [18,19] and the association between LUTS and depression severity of patients clinically diagnosed with depression or neurotic disorders [10,20]. The total score of the IPSS is between 0 and 35. The final score is assigned to the following 4 severity categories: no symptoms (0), mild (1–7), moderate (8–19), and severe (20–35). The IPSS was designed to be self-administered by the patient.

The International Consultation on Incontinence Questionnaire-Short Form (ICIQ-UI SF) was used to assess urinary incontinence because the IPSS does not provide any feedback on this important storage symptom. The ICIQ-UI SF has 4 specific items that assess the burden of urinary incontinence [21]. The questions cover frequency, severity, and overall impact of urinary incontinence. The total score is between 0 and 21. The ICIQ-UI SF may be divided into the following 4 severity categories: slight (1–5), moderate (6–12), severe (13–18), and very severe (19–21) [22]. The ICIQ-UI SF, a self-administered questionnaire, is used widely in routine clinical care of male and female UI patients.

### 2.2. Statistics

Means, standard deviations (SDs), medians, minimum and maximum values (range), and 95% confidence intervals (CI) were used to present descriptive results for continuous data and counts and percent for discrete data. The Shapiro–Wilk test was used to analyze distribution and the Leven (Brown–Forsythe) test was used to investigate the hypothesis of equal variances. To evaluate differences between 2 groups (unrelated variable model), we used the Student’s *t*-test (or Welch test in the absence of variance homogeneity) or Mann–Whitney U test (if the Student’s *t*-test could not be applied or for variables measured on the ordinal scale). The logistic regression model was used to investigate the effect of LUTS or UI on sleep quality in depressed patients regardless of age and sex, making our results comparable to other studies [23]. To establish a link, strength, and direction between variables, correlation analysis was used by calculating Pearson and/or Spearman correlation coefficients. Statistical significance was considered when the *p* value was <0.05. Data analysis was conducted with STATISTICA Software (StatSoft Inc, Tulsa, OK, USA, 2014, ver 12.0).

## 3. Results

### 3.1. Demographic and Clinical Characteristics

Initially, we enrolled 106 subjects, of which 4 patients were excluded owing to incomplete questionnaires. Thus, 102 patients were analyzed. The mean age of our cohort was 46.1 (range: 20–67). There were more women than men. Most of the included patients were employed, had higher education, and were in stable relationships according to patient assumptions (Table 1).

The mean time between diagnosis of depression and inclusion in the study was 10.7 years. For the study cohort, the mean number of hospitalizations related to depression was 2.4 (range: 0–20). We investigated the familial history of depression for 31 individuals. Patients were mostly treated with selective serotonin reuptake inhibitors and serotonin-norepinephrine reuptake inhibitors (Table 2). Only six patients were taking medications occasionally to aid with sleep (zolpidem or zopiclone), and their demographic and clinical characteristics were not statistically different from the rest of the included patients. In addition, we identified only four patients treated for LUTS (one with beta-3 agonist and three with alpha-blockers). Similarly, their demographic and clinical characteristics were not different from the remaining individuals.

Some of our patients had concomitant anxiety (*n* = 19), personality (*n* = 4), obsessive-compulsive (*n* = 3), and eating (*n* = 3) disorders. All these patients met specific ICD-10 criteria for these specific concomitant disorders, and psychiatrists established the diagnoses of all concomitant psychiatric disorders.

### 3.2. Instruments

From analyzing the scores of the Hamilton Rating Scale for Depression (HRSD) questionnaire (mean score: 15.9; range: 1–32), we found that most of our patients experienced mild depression (*n* = 37), then moderate depression (*n* = 29), severe depression (*n* = 20), or were in remission (*n* = 16).

**Figure 1:**
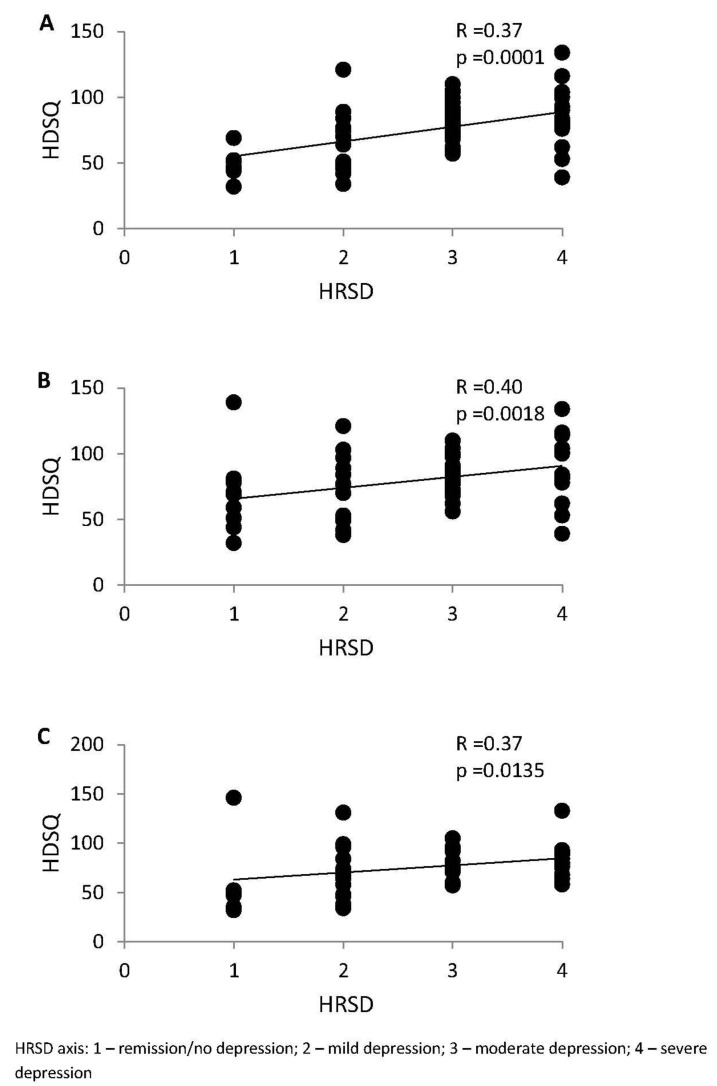
Correlations of depression severity based on HRSD (score in categories) and sleep quality based on HSDQ in the study groups. (**A**) All, (**B**) women, (**C**) men.

The mean score from the Holland Sleep Disorders Questionnaire (HSDQ) was 77.4 (range: 32–146). We found a statistically significant and positive correlation between depression severity and sleep quality (Figure 1A). Further, this relationship was observed in both women and men (Figure 1B,C). We also found significant association between sleep quality and depression severity in groups of patients between 31–40 (R = 0.64, *p* = 0.0061) and 51–60 (R = 0.37, *p* = 0.0209) years of age. We noticed similar trends between sleep quality and depression in other age groups, but these correlations were not statistically significant.

**Figure 2:**
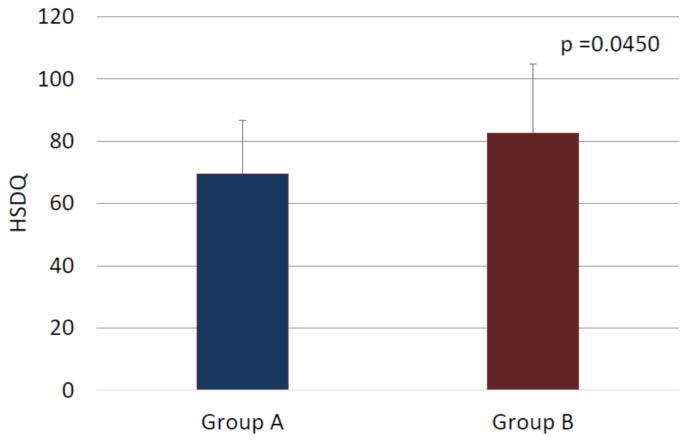
Comparison of depressed patients with mild or no urological symptoms (Group A, *n* = 70) and depressed patients with moderate or severe urological symptoms (Group B, *n* = 32) in terms of the sleep quality based on HSDQ.

The mean score from the International Prostate Symptom Score (IPSS) was 8.1 (range: 0–35). Patients were classified as not symptomatic (*n* = 14), mildly symptomatic (*n* = 56), moderately symptomatic (*n* = 24), and severely symptomatic (*n* = 8). Therefore, most of our patients had LUTS (i.e., 86.3% of patients reported at least mild symptoms based on the IPSS instrument). We found a statistically significant correlation between sleep quality and LUTS severity. A group of depressed patients with moderate or severe LUTS had greater sleep problems than those with mild symptoms or no symptoms (*p* = 0.0450, Figure 2). Table 3 displays comparative characteristic of these two analyzed groups. Post-hoc tests investigating specific items of the IPSS showed that nocturia had the strongest impact on the sleep quality of the included patients. In our cohort, a greater number of nocturia episodes (at least three episodes) led to significantly higher HSDQ scores.

The mean score from the International Consultation on Incontinence Questionnaire-Short Form (ICIQ-UI SF) questionnaire was 7.2 (range: 3–20). In total, 37 patients (36.3%) reported UI (28 females and 9 males). We assigned UI patients to 4 severity categories: slight (15 patients), moderate (19 patients), severe (2 patients), and very severe (1 patient). Most of the UI patients (19 individuals) complained of UI “about once a week or less often.” Seven patients experienced UI “several times a day,” five patients “2 or 3 times a week,” and three patients “about once a day.” Although depressed patients with concomitant UI had higher overall scores from the HSDQ (mean: 85.4, SD: 21.1) compared with those without UI (mean: 77.3, SD: 22.3), the relationship between UI and sleep quality was not statistically significant (*p* = 0.079).

Of the 37 patients with UI, 35 patients complained of at least moderate LUTS as assessed with the IPSS. This patient subgroup reported mostly the frequency (question #2 from the IPSS, mean score: 2.74 ± 1.65), urgency (question #4 from the IPSS, mean score: 2.25 ± 1.68), and occurrence of nocturia (question #7 from the IPSS, mean score: 2.97 ±1.74). Patients also reported, with lower severity, incomplete emptying (question #1 from the IPSS, mean score: 1.05 ± 1.39), intermittency (question #3 from the IPSS, mean score: 1.31 ± 1.56), weak stream (question #5 from the IPSS, mean score: 1.25 ± 1.42), and straining (question #6 from the IPSS, mean score: 0.82 ± 1.13).

We did not find statistically significant associations between sleep quality, incidence or severity of LUTS or UI and psychiatric medications that our cohort used.

### 3.3. Regression Analysis 

With regression analysis, we investigated the effect of depression severity, LUTS, and UI on sleep quality in depressed patients regardless of age and sex (Table 4). We showed that depression severity affects sleep quality. Patients with no depression or mild depression (HRSD 0–16) had better sleep quality than patients with moderate or severe depression (HRSD ≥ 17). Further, the severity of LUTS assessed with the IPSS was associated with decreased sleep quality, no LUTS led to better sleep quality, and severe LUTS led to worse sleep quality. Similarly, worsening of UI decreased quality of sleep; patients with mild UI were characterized by better sleep quality, whereas patients with severe UI were characterized by worse sleep quality.

## 4. Discussion

To our knowledge, our study is the first to analyze the correlation between sleep quality and LUTS in patients with depression. In our cohort of depressed patients, sleep disturbance correlated with LUTS, including UI, regardless of age and sex. The negative impact of LUTS on depressed patient sleep quality that we observed should lead to the re-evaluation of current recommendations on diagnosis of sleep problems for these patients [24,25,26,27]. Patients will benefit when their clinical caregivers are aware of the association between sleep quality and LUTS. Screening for LUTS and UI for depressed patients who report sleep problems is necessary because these disorders and symptoms tend to coexist and may compound the severity of one another. Moreover, presenting a reliable treatment plan for both conditions will ensure that patients receive adequate and careful complex therapy. Strong concerted efforts of diverse specialists are required for this specific patient group because psychiatrists may have limited perception of LUTS in their patients [28]. However, it is not enough that psychiatrists have adequate education about the impact of LUTS and UI on patient management, prognosis, and quality of life. The urology community should also be extra aware of significant associations between different LUTS and sleep quality of depressed patients who are often underdiagnosed and undertreated for urologic disorders [28]. Our study supports these findings because despite the relatively high prevalence and bother of LUTS in our cohort of depressed patients, only few individuals had LUTS-dedicated treatment.

A strength of this study was our evaluation of a homogenous group of patients who all met DSM-5 and ICD-10 criteria for depression. In all cases, psychiatrists confirmed the diagnoses. All included patients were also treated for depression. The study results therefore clearly show the relationship between sleep quality and LUTS (including UI) in this specific patient group. In other analyses of sleep quality and LUTS, investigators included only participants from the general population [4,5,6,7]. Some of these researchers even suggested that they analyzed correlations between depression, sleep quality, and LUTS [15]. However, psychiatrists did not confirm the diagnoses of depression in those studies. Instead, diagnoses were based only on the results of single instruments, sometimes not even standardized measures, to assess depression. The measures that were utilized in general population studies were used only to screen for depression and were not diagnostic, even if they were valid tools to assess symptom load [29]. Therefore, all earlier studies, in fact, analyzed correlations between sleep quality and LUTS in patients from the general population or, at best, in patients with depressive symptoms but not confirmed depression. Thus, we suggest that our study is the first that analyzed the correlation between sleep quality and LUTS, including UI, in a group of reliably diagnosed patients who were treated for depression.

Another strength of our study was the use of the validated scales for assessment of depression severity, sleep quality, LUTS, and UI. These evaluation devices limited the possibility of both under- and overreporting urological and psychiatric symptoms and sleep problems. All used instruments showed good measurement properties in studies conducted in both general and patient specific populations, including depressed patients [30]. Even the largest population-based studies that assessed LUTS-sleep quality correlations rarely used validated instruments, a deficiency which was considered by experts as a significant limitation [4].

LUTS are multifactorial and often need to be considered as urological manifestations of systemic disease processes. A correlation between LUTS and depression has been investigated by multiple studies [30]. These studies have proposed the bidirectional nature of relationship between LUTS and depression. Further, several shared pathways have been considered as mechanisms for the LUTS-depression relationship [30]. First, LUTS reduce quality of life and may lead to embarrassment and poor self-esteem, which negatively affect patient perception as a sign of weakness. This emotional distress caused by LUTS may provoke, exacerbate, or prolong depressive symptoms. Second, some antidepressants and anxiolytics have been presented as risk factors for LUTS [31]. Third, altered concentrations of serotonin and norepinephrine in the central nervous system in both LUTS and depressed patients may represent a biochemical mechanism of coexistence of LUTS with depression [32]. Moreover, increased adrenergic tone and the hypothalamic-pituitary axis have been suggested to mediate the depressive symptoms and LUTS [33]. Finally, even inflammation that is involved in both LUTS and depression pathophysiology has been proposed as a mechanism of action [34].

The connections between LUTS and sleep disorders have been investigated in both cross-sectional and longitudinal population-based studies. In the CAMUS trial, LUTS severity was a risk factor for severe sleep disturbance [7]. Although nocturia was significantly associated with sleep disturbance in the CAMUS cohort, other LUTS were also independent predictors of sleep dysfunction. The REDUCE study showed that men with sleep problems were at higher risk for LUTS if they were asymptomatic at baseline [5]. The NAGAHAMA study presented concurrent findings because baseline sleep disturbance was significantly associated with LUTS cross-sectionally and with worsening LUTS longitudinally [35]. In the NHANES study, sleep disorders were associated with an increased risk of nocturia and daytime LUTS independent of body mass index, diabetes, and a large number of comorbidities [4]. Although these studies have clearly shown the link between LUTS and sleep quality, they have also demonstrated that nocturia may not have been the only significant predictor of sleep disturbance because other LUTS also contributed to impaired sleep patterns. Therefore, LUTS and sleep disorders remain a significant clinical, financial, and quality of life burden.

Our study also alleviates the paucity of available data on the effect of nocturia on sleep quality. It is currently established that one nightly void does not appear to be sufficiently disruptive to cause significant bother for most patients [36]. At least two episodes of nocturia is a threshold for bother that substantially diminishes patient quality of life [37]. Nonetheless, experts suggest that this threshold is not irrefutably established and should not be extrapolated to different patient subpopulations [36]. Our analysis of depressed individuals revealed that at least three episodes of nocturia led to significantly lower quality of sleep in this unique population. We speculate that depressed patients may have greater tolerance for nocturia because of their primary disorder, i.e., depression.

Our study should be viewed in the context of its limitations. First, we acknowledge that the evaluated patients, treated at a single, high-volume academic center, composed a highly selected cohort. Thus, the results may not be fully transferable to clinical practice for all patients treated for depression. Further, our cohort of study participants lacked controls. Including a control group would probably help to investigate whether patients treated for depression are more vulnerable to sleep disorders associated with LUTS than nondepressed individuals. Second, we were not able to assess patients longitudinally. Thus, we do not know how the associations between LUTS and sleep disorders of depressed patients may change with time as LUTS and sleep disorders are dynamic conditions. However, we obtained the data for this study from a prospectively and carefully maintained database, which functioned to constrain the risk of errors and/or omissions. Finally, although our sample size was large enough for powerful statistical analysis, it is possible that with a larger patient group, a significant influence of UI on sleep quality may be determined without adjusting statistical analysis for age and sex. In addition, we could not identify consistent relationships between specific types of sleep disorders from the HSDQ and LUTS or UI. The study possibly lacked adequate power for these specific and detailed analyses. Thus, further prospective studies with larger cohorts and control groups should investigate these important issues.

## 5. Conclusions

This unique study provides data to determine the effects of LUTS, including UI, in depressed patients who experience sleep problems. In our cohort of patients treated for depression, there was a strong correlation between LUTS and poor sleep quality. Therefore, clinicians should be aware of this association and screen depressed patients for both LUTS and sleep problems. This suggested approach would be novel for physicians who care for depressed patients but would lead to thorough, multidisciplinary management.

## Figures and Tables

**Table 1:** Demographics of included patients.

Specification	Total, N (%)
Number of included patients	102 (100%)
Sex	
Male	42
Female	60
Education	
Primary	3
Secondary (including students)	45
Higher	54
Employment status	
Employed	55
Unemployed	13
Pensioners	30
Students	4
Relationship	
Stable relationship/marriage	73
Unstable relationship/marriage	12
Single	17

**Table 2:** Drugs taken by the included patients. SNRIs—serotonin norepinephrine reuptake inhibitors; SSRIs—selective serotonin reuptake inhibitors; TCAs—tricyclic antidepressants; NaSSAs—noradrenergic and specific serotonergic antidepressants; SARIs—serotonin antagonist and reuptake inhibitors.

Drugs	Number of Patients
Antidepressants	
SNRIs	47
SSRIs	46
TCAs	23
NaSSAs	21
SARIs	21
Lithium	14
Others antidepressants	10
Anti-epileptics	
Valproate	23
Lamotrigine	16
Carbamazepine	10
Neuroleptics, first generation	
Phenothiazines	35
Thioxanthenes	13
Butyrophenones	6
Neuroleptics, second generation	
Quetiapine	24
Sulpiride	16
Olanzapine	14
Aripiprazole	8
Other neuroleptics	6
Anxiolytics	
Benzodiazepines	33
Hydroxyzine	10
Buspirone	3

Of note: Other antidepressants—tianeptine, norepinephrine and dopamine reuptake inhibitors (NDRIs), norepinephrine reuptake inhibitors (NRIs), reversible monoamine oxidase inhibitor (RIMAs), agomelatine. Other neuroleptics—risperidone, clozapine, amisulpride.

**Table 3:** Comparative characteristic of depressed patients with mild or no urological symptoms (Group A, *n* = 70) and depressed patients with moderate or severe urological symptoms (Group B, *n* = 32) in terms of the sleep quality based on HSDQ.

	Group A	Group B	*p*-Value
HSDQ			0.0450 ^1^
Mean (standard deviation)	69.4 (17.4)	82.5 (22.4)	
Range	34.0–91.0	38.0–134.0	
Median	72.0	81.5	
95% confidence interval	[59.3;79.4]	[77.1;87.8]	

^1^ U Mann–Whitney.

**Table 4:** Regression analysis of the effect of depression severity, lower urinary tract symptoms (LUTS) assessed with IPSS, and urinary incontinence (UI) assessed with the ICIQ-SF UI on sleep quality in patients with depression regardless of age and sex.

Parameter	*p*-Value
HRSD	**0.0010**
Remission/no depression (0–7)	**0.0119**
Mild depression (8–16)	0.2123
Moderate depression (17–23)	0.2780
Severe depression (≥24)	**0.0193**
HRSD (0–16)	**0.0020**
HRSD (≥17)	**0.0020**
IPSS	**0.0001**
None (0)	**0.0291**
Mild LUTS (1–7)	0.1659
Moderate LUTS (8–19)	0.1306
Severe LUTS (20–35)	**0.0011**
ICIQ-UI SF	**0.0003**
Mild UI (1–5)	**0.0054**
Moderate UI (6–12)	0.1030
Severe UI (13–18)	**0.0043**
Very severe UI (19–21)	0.2865

The bold figures indicate statistically significant results.

## Data Availability

All data generated or analysed during this study are included in this published article.
